# Mucinous Adenocarcinoma of Colon

**Published:** 2010-12-01

**Authors:** Naima Zamir, Soofia Ahmed, Jamshed Akhtar

**Affiliations:** Department of Pediatric Surgery, National Institute of Child Health Karachi, Pakistan

**Keywords:** Colorectum, Mucinous adenocarcinoma, Bleeding per rectum, Child

## Abstract

Bleeding per rectum is a common complaint in pediatric age group and mostly relates to benign conditions. Underlying colorectal carcinoma is a rare cause and carries a poor prognosis. We report two cases of mucinous adenocarcinoma of colon, one in a 9 years old male and other in a female of 12 years. The boy presented with rectal bleeding and increasing constipation of more than three years duration. He had mucinous adenocarcinoma (T3N0MX) of rectosigmoid region and underwent local complete resection of the tumor with colostomy. He also received postoperative chemotherapy and later underwent colostomy reversal. He is tumor free at two years follow up. The girl presented with signs of intestinal obstruction and at colonoscopy a stricture found in descending colon. The tumor was resected and biopsy reported as poorly differentiated mucinous adenocarcinoma with positive mesenteric nodes positive for tumor (T3N2MX). She is on chemotherapy.

## INTRODUCTION

Adenocarcinoma of colon is a rare cause of rectal bleeding in the first decade of life. The reported incidence of this malignancy is about 1.3 -2 per million children. This tumor in pediatric age group shows a different behavior than adults and is associated with poor outcome. Literature is scarce on this condition in children from regional countries. Few cases have been reported from West and India [1,2]. 

Here we report two cases of mucinous adenocarcinoma of colon with short term follow up highlighting various aspects of the condition and management provided. 

## CASE REPORT

**Case 1:** A nine year boy weighing 18 kg, presented with bleeding per rectum, occasionally mixed with mucus, something coming out of anus, and constipation for three years.

There were also complaints of passage of worms (ascaris lumbricoides) through nose and per anum, off and on fever, and significant weight loss during this period. For these problems he remained under treatment elsewhere but did not improve. With the suspicion of rectal polyp patient was referred to surgical department.

Patient belonged to a low socio economic class and had six other siblings. Family history was insignificant though mother had episodes of bleeding per rectum for two years. Child was of thin built with severe anemia. On digital rectal examination a large fixed fungating mass, obscuring the lumen of rectum, felt at the tip of the finger. His hemoglobin was 4.4gm/dl. Patient received multiple blood transfusions. A small incisional, per rectal, biopsy taken from the fungating mass revealed mucinous adenocarcinoma of colon. In the meantime child was further investigated. Serum LDH was 680u/l. Carcinoembryonic antigen (CEA) was not done because funds were not available. Ultrasound abdomen, bone scan and chest x-ray were reported as normal. CT scan pelvis showed a soft tissue mass, 2.8 cm wide and 3 cm long, with rectal wall thickness of 1.2 cm and enlarged para-aortic lymph nodes. Rest of the abdomen and pelvis were reported as normal.

Case was discussed with oncologist and after optimization and thorough counseling regarding the surgical option patient underwent laparotomy. Approximately 5 cm long sessile fungating lesion, situated at recto-sigmoid junction with moderately enlarged mesenteric lymph nodes was found. Rest of the abdomen was free of any metastasis. Tumor was excised along with segmental resection of the gut, with 2 cm proximal and 0.5 cm distal normal tissue beyond the margins of the lesion (Fig. 1). End proximal colostomy with distal Hartmann’s procedure was performed. Postoperatively patient did well except for few episodes of loose motions that was managed conservatively. He was discharged home on 8th postoperative day. Histopathology report showed mucinous adenocarcinoma with clear margins of the resected bowel. Lymph nodes removed at operation were tumor free.

**Figure:**
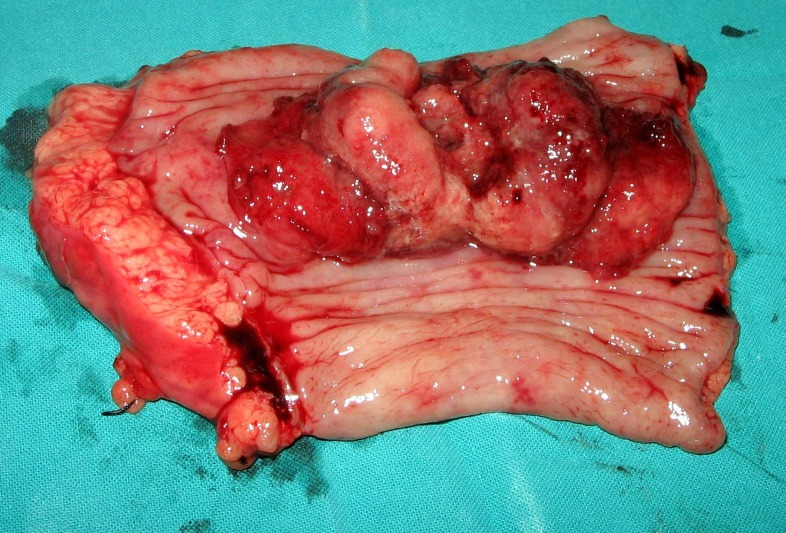
Figure 1: Resected part of colon with fungating mass.

Patient received six cycles of chemotherapy (5 fluorouracil and leucovorin). CT scan repeated three months after completion of chemotherapy, showed rectal wall thickness of one cm with multiple small lymph nodes in the vicinity. Further workup did not show any signs of recurrence or metastasis. Colostomy was closed after one year of initial surgery. Patient is under periodic follow-up and doing well with no recurrence or complications after two and half years of tumor resection.

**Case 2:** A twelve years old female presented with recurrent episodes of abdominal pain, bleeding per rectum, and vomiting since the age of 4 years. For these complaints she received medical treatment in her native city. She was admitted in hospital received multiple blood transfusions but diagnosis could not be established. Her grandfather died of bleeding per rectum and no record of illness was available. 

Patient had colonoscopy six month earlier which showed stricture with suspicion of growth in descending colon (Fig. 2). Biopsy was taken but reported as inconclusive. Later an ultrasound was done which showed a 2.7 cm x 2.2 cm gut related mass. CT scan revealed stricture with bowel thickening at the level of descending colon (Fig. 3). There was no significant para-aortic and mesenteric lymph node involvement. Her CEA was 1.87 ng/ml and bone scan was negative for tumor metastasis. She was then referred to surgical department. She underwent laparotomy and resection of local tumor and regional lymph nodes was done. Divided colostomy was made. Patient did well in post-operative period. Biopsy revealed poorly differentiated mucinous adenocarcinoma of descending colon with margins of resected specimen free of tumor. There was significant lymph nodes involvement. Pathological TNM classification was, T3N2MX. Currently patient had received 2 cycles of chemotherapy (5 fluorouracil and leucovorin). She had single episode of intestinal obstruction during this period and in last follow up had complaints of mild dyspnea, chest and abdominal pain. She is under workup for residual disease and spread of tumor.

**Figure:**
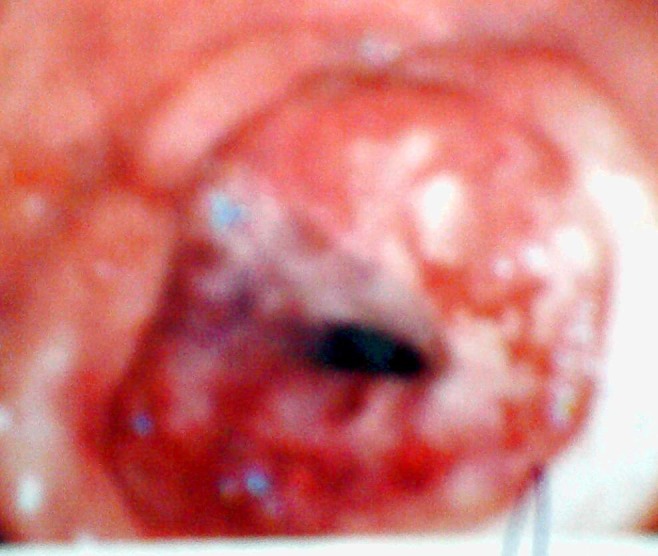
Figure 2: Colonoscopic view of circumferential stricture in descending colon.

**Figure:**
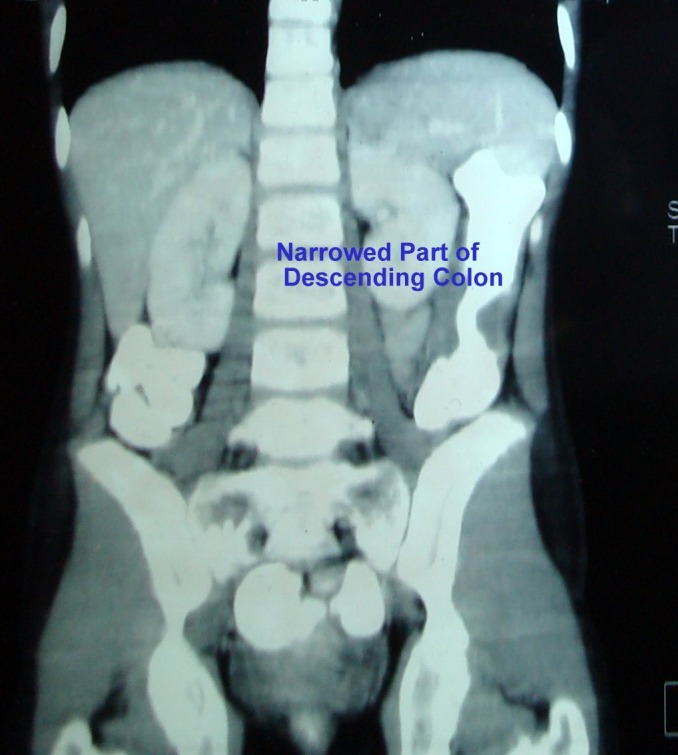
Figure 3: CT scan showing narrowing of descending colon.

## DISCUSSION

Colorectal cancers, though rare, but do occur in pediatric age group. Youngest patient reported was of 9 months. From Pakistan a report of patient above 11 years of age was found on literature search. Based on scanty data available, most of the colorectal cancers in children are reported as sporadic in nature while familial cases do occur [1,3,4]. Though not investigated, both of our patients had relatives with history of bleeding per rectum. The mother of the case one never consulted for per rectal bleeding while grandfather of case 2 had some treatment for his bleeding but no record was available.

Most of the tumors in colon are mucinous adenocarcinoma and bear poor prognosis. In children it is rapidly growing tumor, mostly presenting at advanced stage and five years overall survival is reported to vary between 7 to 12% [2,5]. Usually in pediatric age group patients have an early onset, short duration (usually in months) and are at an advanced stage of disease when seek consult from a physicians. According to a study from United States approximately 86% presented in advanced stages.

Most common presentation is with intestinal obstruction (up to 60% of cases), same occurred in our case 2. Abdominal pain, fever, bleeding per rectum, anemia, and unexplained weight loss are also the common symptoms [2,6,7]. Both of our patients had similar symptoms for three years prior to diagnosis. In case 1 disease was still confined to the bowel wall with no signs of acute or complete intestinal obstruction which is contrary to general concept of colonic adenocarcinoma in childhood. Contrary to Western literature, cases from developing countries showed more of left sided tumors [8]. Similar was the observation in both of our patients, adding evidence to this behavior. Tumor markers like CEA are of no help as documented in literature in pediatric age [2]. This was also true for our case 2, who had normal levels.

These cases are usually missed because most of the children remain under treatment for other benign conditions causing bleeding per rectum or constipation rather than suspicion of malignancy. This could be the reason for misdiagnosis. As no large series is available in pediatric population, treatment is thus based on the adult disease protocol. It depends upon the presentation and stage of disease. Till now surgery remained the mainstay of the treatment. Chemotherapy and radiotherapy are used as adjunct modes for the control of local and distant metastasis and recurrence. Sometimes neo-adjuvant chemotherapy may also be used to shrink the tumor size and facilitate resection. Segmental resection of the gut with end to end anastomosis is the preferred option in early stage of tumor after achieving clear margins. When tumor is non-resectable with local metastasis beyond lymph nodes, incisional biopsy with neoadjuvant chemotherapy is advised. In cases of obstruction and non-resectable tumor, permanent colostomy is preferred. In cases where tumor is resectable but with doubtful margins, as in our case, segmental excision with temporary stoma are advisable followed by chemotherapy [1-3].

Although CT scan, gives a good clue of tumor stage a decision of resectibility can only be made per-operatively. It is therefore important to have a thorough counseling of the patient and parents regarding every possible option of surgical procedure. It is very difficult to accept a permanent stoma for these young children and sometimes they are lost to follow up. It took very long for us to convince the patient’s families for surgical procedures but once they understood the course became easy.

Patient 1 responded to adjuvant chemotherapy very well. Although, he is tumor free for more than two years, recurrence of disease is very much documented in literature [6]. Now it is an important challenge to follow him regularly for early detection of recurrence, if occurs. In second case residual tumor in lymph nodes changed the stage of the disease and chances of abdominal organ system involvement shall remain. Thus a vigilant follow up is mandatory in this case as well.

In conclusion, children with vague abdominal symptoms and bleeding per rectum should not be taken lightly. Digital rectal examination can be of great help if malignancy is suspected. A thorough investigation is required in doubtful cases. Effective counseling of the patient and family is very important. Collaboration between the physicians, surgeons and oncologists for early diagnosis and control of disease, cannot be underestimated.

## Footnotes

**Source of Support:** Nil

**Conflict of Interest:** None declared
